# A practical approach to the spatial‐domain calculation of nonprewhitening model observers in computed tomography

**DOI:** 10.1002/mp.17599

**Published:** 2025-01-08

**Authors:** Gavin Poludniowski, Rebecca Titternes, Daniel Thor

**Affiliations:** ^1^ Department of Nuclear Medicine and Medical Physics Karolinska University Hospital Stockholm Sweden; ^2^ Department of Clinical Science Intervention and Technology Karolinska Institutet Stockholm Sweden; ^3^ Department of Oncology‐Pathology Karolinska Institutet Stockholm Sweden

**Keywords:** computed tomography, model observers, spatial domain

## Abstract

**Background:**

Modern reconstruction algorithms for computed tomography (CT) can exhibit nonlinear properties, including non‐stationarity of noise and contrast dependence of both noise and spatial resolution. Model observers have been recommended as a tool for the task‐based assessment of image quality (Samei E et al., Med Phys. 2019; 46(11): e735‐e756), but the common Fourier domain approach to their calculation assumes quasi‐stationarity.

**Purpose:**

A practical spatial‐domain approach is proposed for the calculation of the nonprewhitening (NPW) family of model observers in CT, avoiding the disadvantages of the Fourier domain. The methodology avoids explicit estimation of a noise covariance matrix. A formula is also provided for the uncertainty on estimates of detectability index, for a given number of slices and repeat scans. The purpose of this work is to demonstrate the method and provide comparisons to the conventional Fourier approach for both iterative reconstruction (IR) and a deep Learning‐based reconstruction (DLR) algorithm.

**Materials and methods:**

Acquisitions were made on a Revolution CT scanner (GE Healthcare, Waukesha, Wisconsin, USA) and reconstructed using the vendor's IR and DLR algorithms (ASiR‐V and TrueFidelity). Several reconstruction kernels were investigated (Standard, Lung, and Bone for IR and Standard for DLR). An in‐house developed phantom with two flat contrast levels (2 and 8 mgI/mL) and varying feature size (1–10 mm diameter) was used. Two single‐energy protocols (80 and 120 kV) were investigated with two dose levels (CTDI_vol_ = 5 and 13 mGy).

The spatial domain calculations relied on repeated scanning, region‐of‐interest placement and simple operations with image matrices. No more repeat scans were utilized than required for Fourier domain estimations. Fourier domain calculations were made using techniques described in a previous publication (Thor D et al., Med Phys. 2023;50(5):2775‐2786). Differences between the calculations in the two domains were assessed using the normalized root‐mean‐square discrepancy (NMRSD).

**Results:**

Fourier domain calculations agreed closely with those in the spatial domain for all zero‐strength IR reconstructions, which most closely resemble traditional filtered backprojection. The Fourier‐based calculations, however, displayed higher detectability compared to those in the spatial domain for IR with strong iterative strength and for the DLR algorithm. The NRMSD remained within 10% for the NPW model observer without eye filter, but reached larger values when an eye filter was included. The formula for the uncertainty on the detectability index was validated by bootstrap estimates.

**Conclusion:**

A practical methodology was demonstrated for calculating NPW observers in the spatial domain. In addition to being a valuable tool for verifying the applicability of typical Fourier‐based methodologies, it lends itself to routine calculations for features embedded in a phantom. Higher estimates of detectability were observed when adopting the Fourier domain methodology for IR and for a DLR algorithm, demonstrating that use of the Fourier domain can indicate greater benefit to noise suppression than suggested by spatial domain calculations. This is consistent with the results of previous authors for the Fourier domain, who have compared to human and other model observers, but not, as in this study, to the NPW model observer calculated in the spatial domain.

## 1 INTRODUCTION

The report of the American Association of Physics in Medicine (AAPM) Task Group 233^1^ recommends the use of task‐based image quality metrics for the evaluation of low‐contrast detectability in computed tomography (CT) systems, stating that: “… task‐based image quality metrics are well suited to characterize and/or compare image quality between imaging conditions in which noise magnitude, noise texture, and/or resolution might be variable.”

Such an approach to image quality assessment requires a well‐specified task, images from patients or a phantom, and a means of evaluating it. Human observers can perform evaluations through, for example, receiver operating characteristics (ROC) and multiple‐alternative forced choice (M‐AFC) experiments. Task‐based evaluation is also possible using model observers, which attempt to predict the performance of humans in such studies, based on mathematical models.^2^ If a model observer is reliable, it can be used to inform the design of new imaging systems, compare the expected performance of different imaging systems, or even optimize imaging protocols—all without the cumbersome and time‐intensive experiments with human readers.

A model observer of fundamental interest is the nonprewhitening matched filter (NPW), which was also selected as an exemplar by Task Group 233. This observer has been shown to correlate well with human observers for tomographic images exhibiting correlated noise,^3^, ^4^, ^5^ although quantitative prediction of results from human studies requires the introduction of additional factors such an eye filter and internal noise, or an empirical efficiency factor.^6^ The signal‐to‐noise (SNR) of the NPW decision variable is considered the figure of merit, and associated with the detectability index, *d′*.

The starting point in expositions of the NPW filter concept is its definition in the spatial domain.^2^ Despite this, it is typically calculated in the frequency domain because of its convenient representation in terms of the task transfer function (TTF) and noise power spectrum (NPS), when the system exhibits stationary noise. There are well‐documented methodologies for determining these two quantities, as summarized in Samei et al.^1^ Once they have been determined, the detectability index of an arbitrary feature can be calculated, assuming that the TTF is available for the particular contrast level to be evaluated. However, accurately determining the TTF and NPS is not without practical challenges.^7^, ^8^ Further, their application assumes that the determined noise and resolution properties accurately reflect those at the precise location of interest both when the feature signal is present and absent—a requirement complicated by the behavior of modern reconstruction algorithms.

In this work, we demonstrate an alternative approach of directly estimating the NPW observer in the spatial domain for features embedded in a phantom, using repeated scanning and simple region‐of‐interest (ROI) placements. No additional repeats of scans were necessary above those that would be necessary for reliable TTF determination. In addition, a formula for the expected uncertainty for a given number of slices and repeat scans is presented. Although the concept of spatial domain calculations for such observers is hardly new,^9^, ^10^ we feel that it is timely to put renewed emphasis on the approach in CT with the outline of a practical implementation. Note that the methodology makes no explicit determination of the noise covariance matrix, as would be essential for spatial domain calculation of a prewhitening matched filter or Fisher‐Hotelling observer^2^ and has also, in some cases, previously been relied on for NPW observer determinations.^10^ It is worth highlighting that several studies in the field of CT have compared the performance of NPW‐based observers in the Fourier domain with spatial‐domain channelized Hotelling observers (CHOs),^5^, ^11^ or even NPW‐based observers in the spatial domain with the CHO.^12^ Relevant comparisons between spatial and Fourier domain calculations of the same observer appear to be lacking.

The approach presented here can be seen as complimentary to the frequency domain methodology. While it cannot be used to calculate the detectability for an arbitrary feature, it is closer to a true experimental determination for a synthetic feature realized in a phantom geometry. It can also be used for validating the assumptions made in frequency domain calculations for modern reconstruction algorithms—whether iterative or based on deep learning—for which the applicability of a Fourier‐based methodology is in question.^11^, ^13^ It is worth noting that task‐based evaluation of deep learning reconstruction (DLR) has received recent attention, due to observations that assessments of noise suppression based on some traditional global fidelity metrics have proved to correlate poorly with both model and human observer performance.^14^, ^15^, ^16^

## 2 MATERIALS AND METHODS

### 2.1 Model observer framework

#### 2.1.1 Linear model observers and detectability

For an image, a general formulation of the linear decision variable of a model observer is,

(1) $$L_{\mathrm{MO}}\left(i\right)=w^{T}\;i$$

where i is an image represented as a vector, w is a template vector for a task, and T indicates the matrix transpose. Different choices of template provide various mathematical observers, which can include the prewhitening matched filter (PW), and the NPW and its variants.^2^, ^6^

A greater SNR for a decision variable implies a greater probability of making a correct decision between two hypotheses. The SNR squared is defined as,

(2) $${\mathrm{SNR}}^{2}=\frac{E{\left[{\left.L_{\mathrm{MO}}\right|}_{h=2}-{\left.L_{\mathrm{MO}}\right|}_{h=1}\right]}^{2}}{\frac{1}{2}\mathrm{Var}\left[{\left.L_{\mathrm{MO}}\right|}_{h=1}\right]+\frac{1}{2}\mathrm{Var}\left[{\left.L_{\mathrm{MO}}\right|}_{h=2}\right]},$$

where E[…] indicates the expected value, Var[…] is the variance and h=1,2 indicates the two hypotheses. The numerator and denominator represent the squares of “signal” and of “noise,” respectively, for the decision variable. Subsequently, such noise will be referred to as decision‐level noise, to distinguish it from the image noise properties on which it depends, such as pixel standard deviation and the noise covariance matrix.

The so‐called detectability index, *d’*, is defined^17^, ^18^

(3)

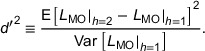




Under this definition, equality between *d’* and SNR will hold when the noise properties in an image are independent of whether a signal is present or absent.

#### 2.1.2 The nonprewhitening matched filter

We consider a situation where a known feature is either absent ($i_{h=1}$) or present ($i_{h=2}$) on a known background, that is, a signal known exactly/background known exactly scenario. We define,

(4) $$\Delta \;\bar{i}=E\left[i_{h=2}-i_{h=1}\right]$$

for the expected value of the difference image, or average over an infinite ensemble of images. This is precisely the definition of the template vector for the NPW observer.^2^ The NPW template (or filter) is “matched” in the sense that it is matches the expected difference in images under the two hypotheses. This mathematically represents an observer who, perceptually, cannot prewhiten (decorrelate) the noise in the image. The associated decision variable for a specific image instance is,

(5) $$L_{\mathrm{NPW}}\left(i\right)=\Delta {\bar{i}}^{T}\;i.$$

The SNR for the model observer is

(6) $${\mathrm{SNR}}_{\mathrm{NPW}}^{2}=\frac{{\left(\Delta {\bar{i}}^{T}\;\Delta \bar{i}\right)}^{2}}{\Delta {\bar{i}}^{T}\;n_{i}\;\Delta \bar{i}},$$

where $n_{i}$ is the noise covariance matrix averaged over both hypotheses.

Under certain assumptions, SNR_NPW_ can also be conveniently represented in the Fourier domain. If we assume a linear system with additive external noise then,

(7) $$i_{h}=t\;f_{h}+\varepsilon ,$$

where $f_{h}$ is the input function under a hypothesis *h*, t is the spatial resolution transfer, and ε is the noise. If we can assume shift‐invariance, then we may utilize the Fourier domain and write,

(8)

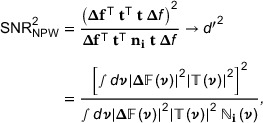

where ν is the 2D spatial frequency, T is called the TTF, $N_{i}$ is the NPS, and $\Delta f=f_{h=2}-f_{h=1}$ is the input function difference under the two hypotheses. Here, the use of large case “double‐struck” symbols indicates the two‐dimensional Fourier transform of a small case quantity. It has become customary to assume (quasi‐)stationarity and calculate the NPW observer using Equation (8), with a theoretical task function and phantom determinations of the TTF and NPS.^1^, ^4^, ^5^, ^11^, ^18^

#### 2.1.3 Inclusion of an eye filter

The NPW observer does not consider the varying sensitivity of the human eye to differing spatial frequencies. To account for this, an eye filter has been applied with some success,^4^, ^5^, ^6^ typically borrowing the form from contrast sensitivity curves determined with sinusoidal gratings. The result is the nonprewhitening matched filter observer with eye filter (NPWE). The eye filter can be considered to act upon an image following the spatial resolution transfer function and addition of external noise:

(9) $$i_{h}^{e}=e\;i_{h}=e\left(t\;f_{h}+\varepsilon \right),$$

with,

(10) $$L_{\mathrm{NPWE}}\left(i\right)=L_{\mathrm{NPW}}\left(e\;i\right)=\Delta {\bar{i}}^{T}\;e^{T}\;e\;i.$$

This translates into the following SNR:

(11)

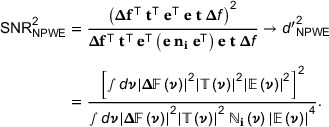




In this work, a circularly symmetric eye filter due to Burgess et al.^19^ will be used, defined as,

(12) $$E\left(\nu \right)=\rho^{a_{1}}\;\exp\left(-\frac{a_{1}}{a_{2}}{\left(\frac{\rho }{\rho_{\mathrm{peak}}}\right)}^{a_{2}}\right),$$

where $a_{1}$ = 1.3 and $a_{2}$ = 2 and the eye filter has a peak sensitivity at $\rho_{\mathrm{peak}}=4$ cycles/degree. This filter was selected only for the purposes of demonstration, but has previously been applied to detectability in CT.^20^ Since the Fourier variable ν is typically expressed in units of reciprocal length at the real‐life scale of an imaged object,

(13) $$\rho =\frac{\pi \;\mathrm{FOV}\;R}{180\;D}\left|\nu \right|,$$

where FOV is the reconstruction field‐of‐view, *R* is the viewing distance and *D* is the display size on a monitor. Values of *R* = 475 mm and *D* = 350 mm will be assumed.^5^

Under typical viewing conditions, the eye filter has the effect of reducing the extent to which detectability increases with feature size. It is noteworthy, however, that the eye filter strongly suppresses very low spatial frequencies in the noise as well as in the signal. This is particularly relevant in scenarios where the NPS is non‐zero at zero spatial frequency (e.g., in radiography and fluoroscopy).^21^

#### 2.1.4 Spatial domain calculation

For a feature embedded in a phantom, the SNR_NPW_ can be estimated using a set of *n* repeat scans. To do so, we must first determine the template (matched filter). If the input signal and contrast are known, as well as the system spatial transfer, then it can be calculated. Alternatively, it can be estimated by averaging images with the feature present and subtracting an estimate of the background.

Here, we will approximate a background subtracted image as,

(14) $$i^{s}=i-b1,$$

where *b* is a scalar background value and **1** is the identity matrix. A uniform background is assumed, determined as the mean pixel value in an ROI close to each feature. In general, however, b1 could be replaced with a spatially varying background image representing the average signal absent case. The use of Equation 14 leads to a template,

(15) $$\Delta \bar{i}\approx \langle i_{h=2}^{s}\rangle ,$$

where the ⟨…⟩ brackets indicate an average over a finite ensemble of images.

The decision variable for a particular image instance can be estimated as,

(16) $$L_{\mathrm{NPW}}\approx {\langle i_{h=2}^{s}\rangle }^{T}\;i^{s}.$$

The mean signal for the decision variable can then be estimated as,

(17) $$\begin{array}{ccc} S & = & E\left[{\left.L_{\mathrm{NPW}}\right|}_{h=2}-{\left.L_{\mathrm{NPW}}\right|}_{h=1}\right]\\ & & \approx {\langle i_{h=2}^{s}\rangle }^{T}\langle i_{h=2}^{s}\rangle =\sum_{j\in \mathrm{ROI}}{\langle i_{j,h=2}^{s}\rangle }^{2}, \end{array}$$

where for clarity we have expanded the vector multiplication as a summation over the pixels in an ROI covering the feature. The quantity $i_{j,h=2}^{s}$ corresponds to the *j*th pixel value in a background‐subtracted image, under a signal present hypothesis (*h* = 2).

The calculation of the variance of the decision variable does not require the determination of the covariance matrix, if it is based on a set of sampled images.^9^ The variance (i.e., the square of the decision‐level noise, *N*) is the average of the variances under the two hypotheses, that is,

(18) $$\begin{array}{ccc} N^{2} & & \approx \frac{1}{2}\mathrm{Var}\left[{\langle i_{h=2}^{s}\rangle }^{T}i_{h=1}^{s}\right]+\frac{1}{2}\mathrm{Var}\left[{\langle i_{h=2}^{s}\rangle }^{T}i_{h=2}^{s}\right]\\ & & =\frac{1}{2}\;\mathrm{Var}\left[\sum_{j\in \mathrm{ROI}}\langle i_{j,h=2}^{s}\rangle i_{j,h=1}^{s}\right]\\ & & +\frac{1}{2}\mathrm{Var}\left[\sum_{j\in \mathrm{ROI}}\langle i_{j,h=2}^{s}\rangle i_{j,h=2}^{s}\right] \end{array}$$

The scalar quantity ${\left\langle i_{h=2}^{s}\right\rangle }^{T}i_{h=1}^{s}$ or ${\left\langle i_{h=2}^{s}\right\rangle }^{T}i_{h=2}^{s}$ can therefore be calculated for each image instance in a set and the variances in values calculated over the set of images. Ideally, the quantities would be estimated at the same spatial position in different images, using two phantoms that are identical except for the presence or absence of a feature. More practically, the same position in the axial plane might be used in differing image slices or different positions in the same slice (assuming some degree of noise stationarity or symmetry). Note that if the noise is contrast‐independent,

(19) $$N^{2}=\mathrm{Var}\left[{\langle i_{h=2}^{s}\rangle }^{T}i_{h=1}^{s}\right]=\mathrm{Var}\left[{\langle i_{h=2}^{s}\rangle }^{T}i_{h=2}^{s}\right].$$

The assumption above is made in the Fourier domain approach to calculations, as the NPS is determined for the signal‐absent condition. It need not, however, be assumed for the spatial domain determinations. Putting all this together, we may write,

(20) $$\begin{array}{ccc} & & {\mathrm{SNR}}_{\mathrm{NPW}}^{2}\left[i_{h=1},i_{h=2}\right]\\ & & =\frac{{\left(\sum_{j\in \mathrm{ROI}}{\langle i_{j,h=2}^{s}\rangle }^{2}\right)}^{2}}{\frac{1}{2}\mathrm{Var}\left[\sum_{j\in \mathrm{ROI}}\langle i_{j,h=2}^{s}\rangle i_{j,h=1}^{s}\right]+\frac{1}{2}\mathrm{Var}\left[\sum_{j\in \mathrm{ROI}}\langle i_{j,h=2}^{s}\rangle i_{j,h=2}^{s}\right]}. \end{array}$$

The equivalent result for the NPWE observer can be found immediately by recognizing that,

(21) $${\mathrm{SNR}}_{\mathrm{NPWE}}^{2}\left[i_{h=1},i_{h=2}\right]={\mathrm{SNR}}_{\mathrm{NPW}}^{2}\left[e\;i_{h=1},e\;i_{h=2}\right].$$

That is, we can calculate the SNR for the NPWE observer by pre‐filtering the CT images by the eye filter and then calculating the NPW observer. Note that although treating the response of the human eye as a filter in the Fourier domain has its own limitations,^19^, ^21^, ^22^ this is a separate issue from the properties of a CT reconstruction. The applicability of an eye filter is not dependent on whether a CT reconstruction algorithm exhibits non‐linearity.

Because the detectability index is commonly used when applying model observers to medical images,^1^ the notation $d_{\mathrm{SNR}}\left(\equiv \mathrm{SNR}\right)$ will be used subsequently, without necessarily assuming the equality of variances under signal absent and present conditions implicit in our definition of *d’*. For Gaussian distributed decision variables, even without equality of variances, $A_{z}=\phi \left(d_{\mathrm{SNR}}/\sqrt{2}\right)$, where *A_z_* is the area‐under‐the curve of the receiver operating characteristics (ROC) curve and ϕ is the cumulative standard normal distribution.^2^, ^17^

#### 2.1.5 Inclusion of internal noise

Although the inclusion of an eye filter degrades detectability and can improve agreement with trends in human observers, quantitative agreement with human efficiency often requires further additions to the observer model. A common approach is the introduction of internal noise.^22^, ^23^, ^24^ This represents noise introduced into the observation process, internal to the observer, in addition to the external noise in the displayed image itself. As this can have contributions ranging from the imperfect sensitivity of cone cells in the retina to that of the noise on the decision‐making process itself, there is not a unique way to incorporate internal noise. This has contributed to many different implementations being proposed, some justified purely empirically.^4^, ^25^, ^26^, ^27^

Broadly, contributions to internal noise can be classified as either fixed (independent of external noise) or induced (dependent on the external noise).^28^ The former may be due to photon or neural noise^22^ and dominate when the external noise is very low (i.e., low image noise or wide window width for display). The origin of the induced component of internal noise is less clear but can be assumed to dominate in most viewing conditions for radiological images. Due to the variety of approaches, we will not attempt to delineate all the possibilities for including internal noise in Fourier and spatial domain calculations. However, we will outline one prominent approach.

According to Eckstein et al.,^18^ a robust way to inject internal noise into a model observer is in the decision variable itself. When the noise is induced, this can be achieved as follows:

(22) $$L_{\mathrm{MOI}}\left(i\right)=L_{\mathrm{MO}}\left(i\right)+\varepsilon_{\mathrm{int}}.$$

where $L_{\mathrm{MOI}}$ is the decision variable for a model observer with internal noise added and $\varepsilon_{\mathrm{int}}$ is a zero‐mean Gaussian random variable with a variance proportional to the external noise of $L_{\mathrm{MO}}$ (i.e., $\mathrm{Var}\left[\varepsilon_{\mathrm{int}}\right]=\beta \mathrm{Var}\left[L_{\mathrm{MO}}\right]$ where β is a constant of proportionality). In the case of Gaussian‐independent responses, this provides a simple result,

(23) $${\mathrm{SNR}}_{\mathrm{MOI}}^{2}=\frac{1}{1+\beta }\;{\mathrm{SNR}}_{\mathrm{MO}}^{2},$$

which is consistent with some empirical observations.^28^ In this implementation, therefore, the inclusion of internal noise can be applied as a post‐hoc scaling step, regardless of whether the detectability is calculated in the Fourier or spatial domain. A value of β=1 will be assumed in the remainder of this work for both the NPW and NPWE cases. We note, however, that the parameter β will vary between individuals and with model observer choice. Values in the literature range from around 0.6 up to about 10, with the larger values typically required when the NPW observer is used without an eye filter.^3^, ^6^, ^28^

### 2.2 Evaluation of framework

#### 2.2.1 Phantom and setup

**FIGURE 1:**
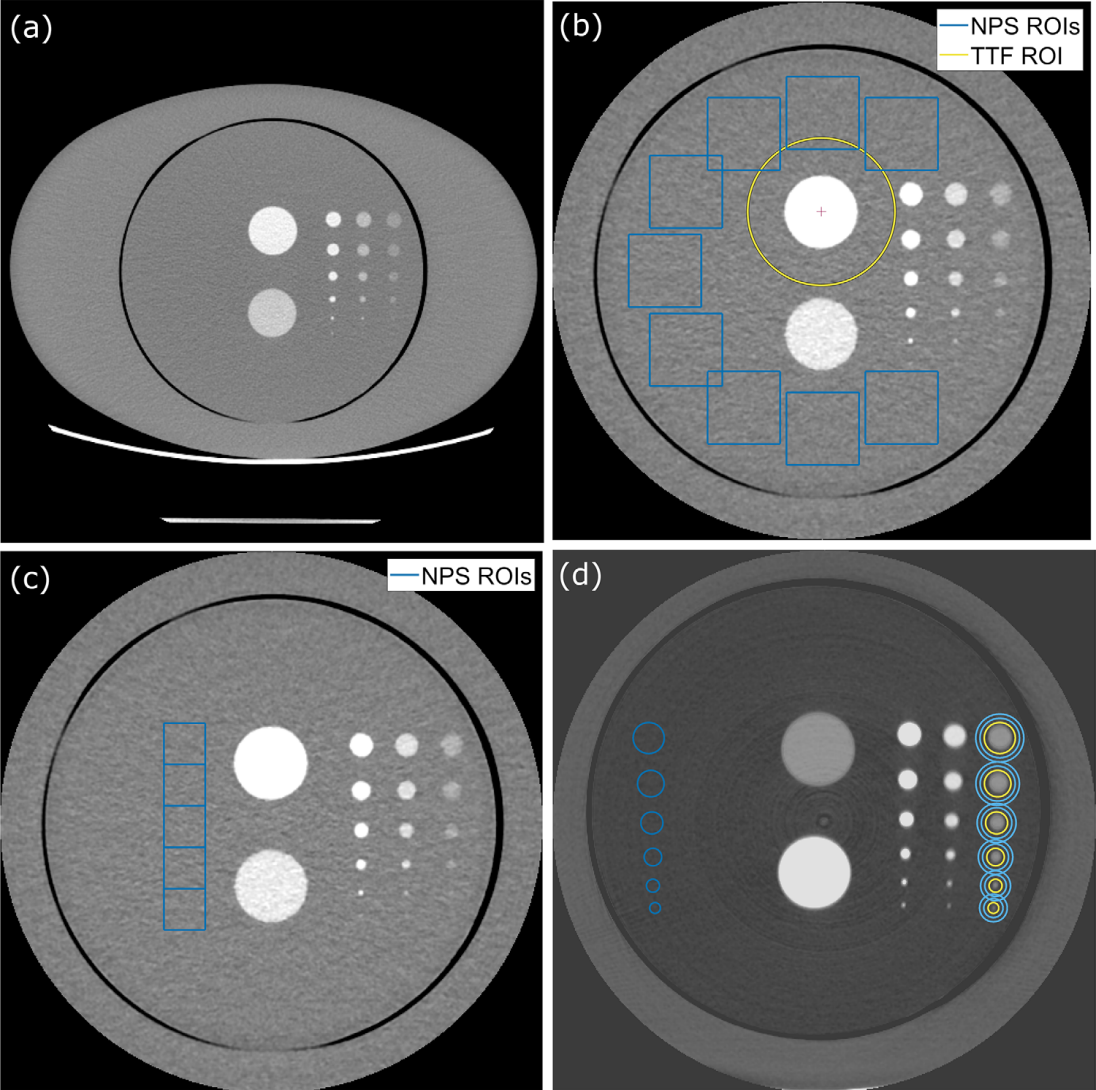
(a) An axial CT image of the phantom with a field‐of‐view (FOV) large enough to encompass the expansion ring. (b) A CT image with a reduced FOV (240 mm); the regions‐of‐interest (ROIs) used to determine the “peripheral” noise power spectrum (dark blue squares) and task transfer function (yellow circle) are illustrated. (c) An image with an illustration of more centrally placed ROIs used for determining the “central” NPS. (d) An axial CT image averaged over repeats. The ROIs used in the study for spatial domain calculations are illustrated; the “feature” ROIs (or ROI_2_) are depicted by yellow circles (right) and the “noise” ROIs (or ROI_1_) by dark blue circles (left). The annuli formed by the light blue circles (right) are those used to determine the average background CT number (“background” ROIs or ROI_b_).

To exemplify the proposed methodology and compare Fourier and spatial domain calculations, CT scans acquired in a previous study were used.^29^ The phantom represented an adult abdomen and consisted of a custom‐manufactured cylindrical section of 200 mm diameter made of solid water substitute, placed inside an elliptical extension ring (CTP579‐15 body annulus, The Phantom Laboratory, Salem, New York, USA) of dimensions 250 mm x 350 mm x 150 mm (H x W x L). The phantom is illustrated by an axial CT slice in Figure 1a. The central disk was 50 mm thick and had a set of drilled cavities of 45 mm depth with diameters of 1, 2, 4, 6, 8, or 10 mm. Two larger features (32 mm diameter) were present in the center, consisting of an additional cavity and a bone insert (not used in the current study). The cavities could be filled with varying concentration of iodine, created by dilution of 350 mgI/mL contrast media (iohexol) by purified water. To better represent realistic scanning conditions, two additional cylinders of the same dimensions as the central section were placed either side of it, so that the entire phantom had a 150 mm thickness along the z‐axis.

The smaller cavities were filled with 2, 4, and 8 mgI/mL of contrast media, with decreasing contrast from left to right as illustrated in Figure 1a. The larger central cavity was filled with either 2 or 8 mgI/mL of contrast agent, depending on the set of image acquisitions. We note that the iodine concentrations investigated in this study range from the relatively low enhancement that might be observed in the liver parenchyma in the venous phase (2 mgI/mL)^30^ up to a rather high value (8 mgI/mL).

#### 2.2.2 Scanning and reconstruction

Single‐energy scans were acquired in axial mode with a Revolution CT scanner (GE Healthcare, Waukesha, Wisconsin, USA) and tube potentials of either 80 or 120 kV. Images were reconstructed with a field‐of‐view (FOV) of 240 mm and a slice thickness of 5 mm. Two dose levels were acquired in each case (CTDI_vol_ of 5 and 13 mGy) and scans were reconstructed with ASiR‐V iterative reconstruction (0%, 50% and 100%) for three kernels (Standard, Lung, and Bone). For the Standard kernel, additional reconstructions using the vendor's TrueFidelity deep learning‐based reconstruction (DLR) algorithm were also possible (low, medium, and high strengths).^31^ ASiR‐V 0% is assumed to represent filtered backprojection (FBP), at least at high dose levels,^29^, ^32^ and used as a reference where assumptions of stationarity are expected to hold best. Reconstructions were performed in situ on the scanner. The scans were repeated without table motion or phantom replacement, with the number of repeats depending on the contrast media concentration in the central cavity and the dose level, such that sufficient averaged slice contrast‐to‐noise (> 15) was achieved for determination of the TTF for all reconstructions with the standard kernel.^8^, ^29^ The number of repeats for data presented in this work were *n* = 26 (5 mGy and 8 mgI/mL), *n* = 42 (13 mGy and 2 mgI/mL), and *n* = 110 (5 mGy and 2 mgI/mL).

The dose levels for the acquisitions (see CTDI_vol_ values) correspond to the lower and high range for abdominal examinations, as exemplified by diagnostic reference levels in the authors’ country‐of‐residence.^33^

#### 2.2.3 Implementation of d_SNR_ calculations

Model observer calculations were made with a fixed number of image slices (*m* = 5) and for the variable number of repeat scans available (*n* = 26–110). Calculations of *d*_SNR_ in the Fourier domain (i.e., *d’*) followed well‐established methodologies and was implemented in MATLAB (MathWorks, Natick, Massachusetts, USA), as described in our earlier work.^29^ The ROIs used for determining the NPS and TTF are depicted in Figure 1b (dark blue squares and yellow circle, respectively). It is worth noting that the positions of the NPS ROIs are on the periphery of the inner section of the phantom. While this is a reasonable representation of the locations for the column of low contrast features, the high contrast features are more centrally positioned. To investigate the possible effects of NPS ROI location, more centrally located ROIs were also used for an alternative “central” NPS (see Figure 1c).

Implementation of calculations in the spatial domain was also performed in MATLAB. The ROIs used are displayed in Figure 1d. The yellow circles (right) constitute the feature ROIs for the signal present case (ROI_2_) and were slightly larger than the feature in each case. The surrounding light blue annuli (ROI_b_) provided local estimates of background values (i.e., *b*) for subtraction from the ROI images. The dark blue circles (left) provided noise ROIs in the left‐right mirror positions (ROI_1_), for estimation of noise in the signal absent cases. As the phantom shape has left‐right symmetry, it was assumed that this would represent the signal‐absent condition, even when noise characteristics depart from stationarity. A step‐by‐step algorithm for the implementation is presented in Appendix A.

All determinations of *d*_SNR_ included an internal noise parameter of β=1 (see Equation 23). Agreement between Fourier and spatial domain estimates of *d*_SNR_ were assessed using the normalized root mean square discrepancy (NRMSD), defined as the RMS discrepancy normalized by the range in *d*_SNR_ values.

#### 2.2.4 Evaluation of uncertainty and bias in d_SNR_

As the spatial domain methodology involves a finite number of repeat scans and slices for the calculations, there will be residual uncertainties, and, potentially, bias. To evaluate the magnitudes of these, simple bootstrap simulations were conducted in MATLAB using sampling with replacement. Scenarios of *m = *1–5 (slices) and *n = *10–110 (repeats) were simulated with 2000 resamples, based on a data set with 5 slices and 110 repeat scans (5 mGy and 2 mgI/mL; ASiR‐V 0%). Uncertainty was estimated from the standard deviation of bootstrapped *d*_SNR_ values. Bias was estimated as the difference between the mean bootstrapped value for a given (*m*, *n*) combination, and the value determined with the entire original data set.^34^

The bootstrapped uncertainties were compared with theoretical results based on the assumption of treating different slices and repeats as independent samples (see Appendix B):

(24) $$u=\sqrt{\frac{1}{\left(1+\beta \right)}\frac{1}{nm}+\frac{1}{4}\frac{1}{\left(n-1\right)m}d_{\mathrm{SNR}}^{2}},$$

where *u* is the uncertainty on $d_{\mathrm{SNR}}$.

## 3 RESULTS

Results for the noise magnitude, noise power spectra, and TTFs are published elsewhere (for the Standard reconstruction kernel)^29^ and will not be reproduced here. Instead, the presentation will be limited to estimations of *d*_SNR_. Displayed error bars correspond to two standard deviations (±2u), as calculated using Equation 24. It is worth noting that although error bars are only displayed for the spatial domain determinations, the Fourier domain calculations also have (undetermined) uncertainties.

**FIGURE 2:**
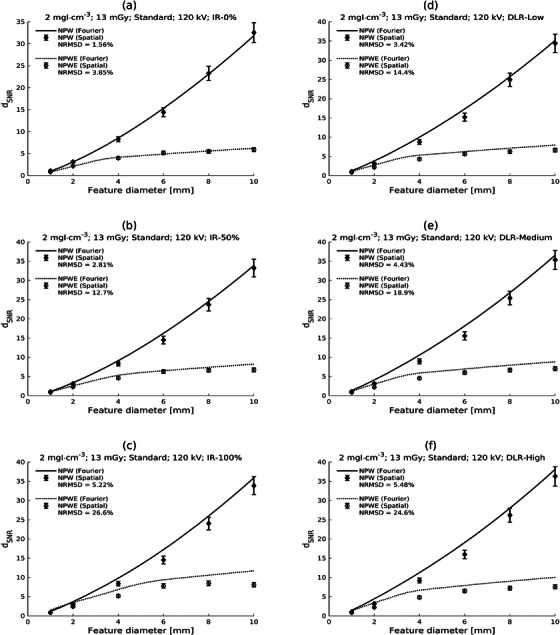
Plots of generalized detectability index (d_SNR_) against feature diameter for Fourier and spatial domain calculations for low contrast (2 mgI/mL) and high dose (CTDI_vol_ = 13 mGy) with the 120 kV protocol and the Standard reconstruction kernel. Results are shown for different reconstructions: ASiR‐V 0%, 50%, and 100% [AR‐0, AR‐50, AR‐100; parts (a)‐(c), respectively] and TrueFidelity Low, Medium, and High [TF‐L, TF‐M, TF‐H; parts (d)‐(f), respectively]. Calculations for the nonprewhitening (NPW) and the nonprewhitening with eye filter (NPWE) observers are shown.

**FIGURE 3:**
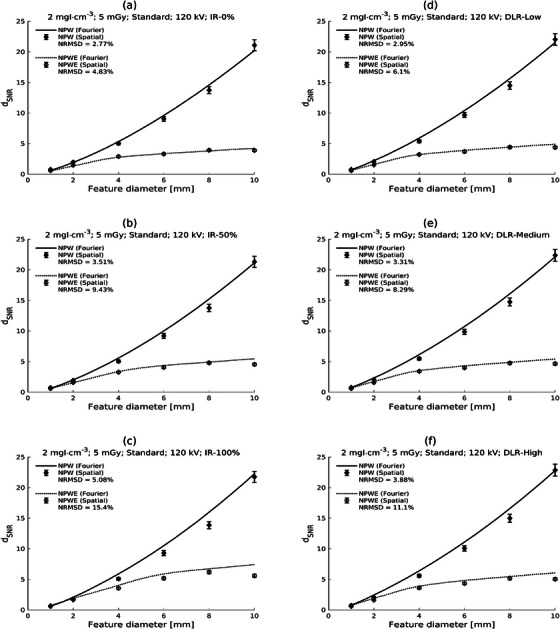
Plots of generalized detectability index (d_SNR_) against feature diameter for Fourier and spatial domain calculations for low contrast (2 mgI/mL) and low dose (CTDI_vol_ = 5 mGy) with the 120 kV protocol and the Standard reconstruction kernel. Results are shown for different reconstructions: ASiR‐V 0%, 50%, and 100% [AR‐0, AR‐50, AR‐100; parts (a)‐(c), respectively] and TrueFidelity Low, Medium, and High [TF‐L, TF‐M, TF‐H; parts (d)‐(f), respectively]. Calculations for the nonprewhitening (NPW) and the nonprewhitening with eye filter (NPWE) observers are shown.

The predictions of *d*_SNR_ for the low‐contrast (2 mgI/mL), high‐dose (CTDI_vol_ = 13 mGy), and the 120 kV protocol with the Standard kernel are shown in Figure 2a–f. Each subfigure shows the NPW and NPWE predictions in the spatial and Fourier domains for a particular reconstruction (ASiR‐V 0%, 50%, or 100% or TrueFidelity low, medium, or high). The corresponding figures for the low dose level (CTDI_vol_ = 5 mGy) are presented in Figure 3a–f. Of note is the close correspondence of spatial and Fourier domain calculations for ASiR‐V 0% reconstructions, with the disagreement remaining reasonable but increasing as the iterative strength or DLR noise‐suppression level is increased (see the NRMSD values in the figures). Discrepancies appear more severe with the inclusion of the eye filter.

**FIGURE 4:**
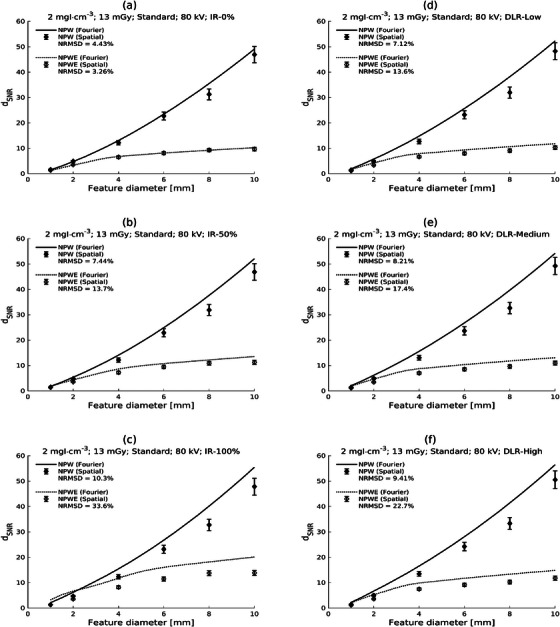
Plots of generalized detectability index (d_SNR_) against feature diameter for Fourier and spatial domain calculations for low contrast (2 mgI/mL) and high dose (CTDI_vol_ = 13 mGy) with the 80 kV protocol and the Standard reconstruction kernel. Results are shown for different reconstructions: ASiR‐V 0%, 50%, and 100% [AR‐0, AR‐50, AR‐100; parts (a)‐(c), respectively] and TrueFidelity Low, Medium, and High [TF‐L, TF‐M, TF‐H; parts (d)‐(f), respectively]. Calculations for the nonprewhitening (NPW) and the nonprewhitening with eye filter (NPWE) observers are shown.

The equivalent results for the 80 kV protocol and high doses are shown in Figure 4a–f. Similar trends are observed as for 120 kV, albeit with somewhat greater discrepancies between the Fourier and spatial domain calculations for reconstructions other than ASiR‐V 0%.

**FIGURE 5:**
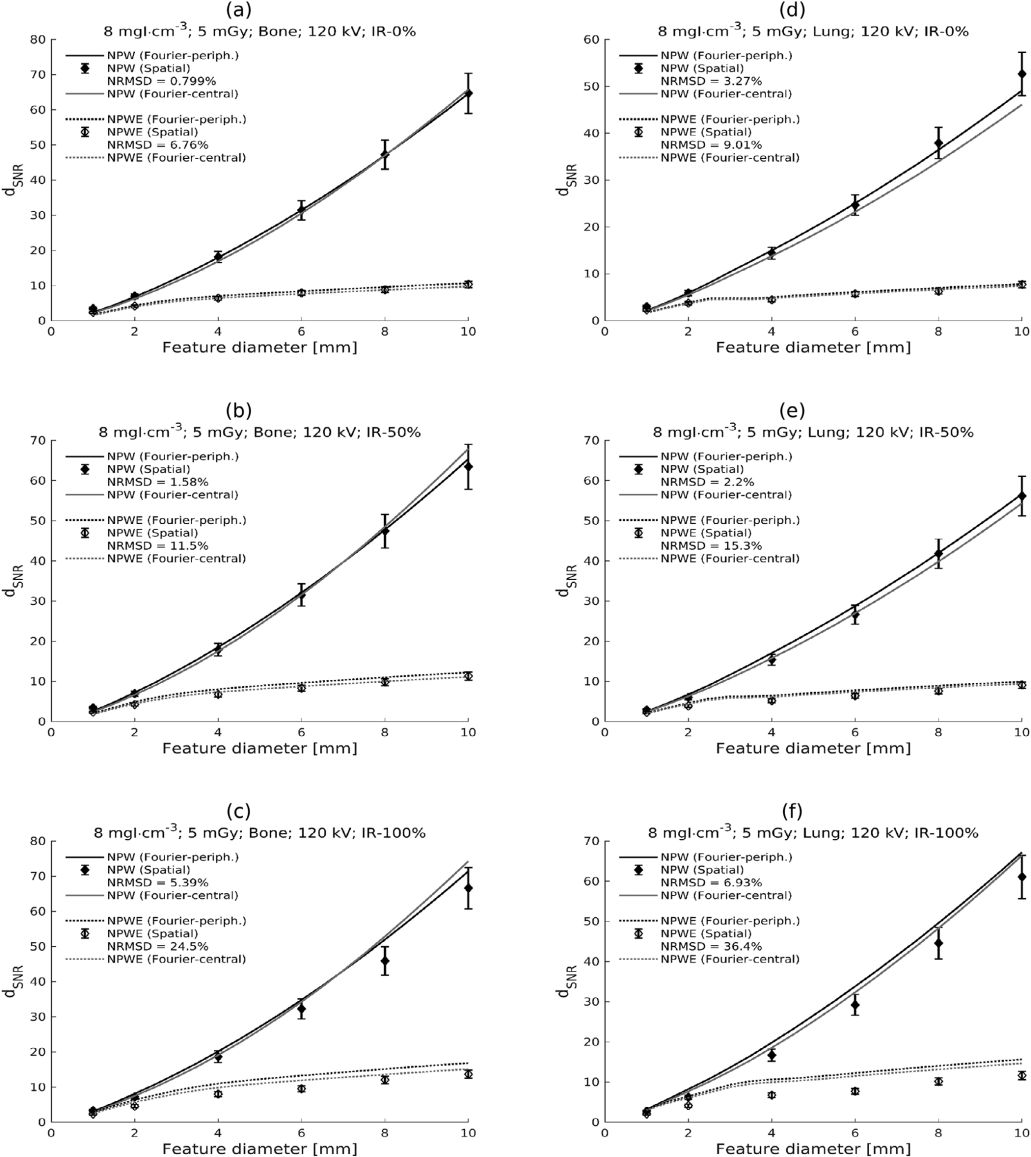
Plots of generalized detectability index (d_SNR_) against feature diameter for Fourier and spatial domain calculations for high contrast (8 mgI/mL) and low dose (CTDIvol = 5 mGy) with the 120 kV protocol and the bone and lung reconstruction kernels. Results are shown for different reconstructions: ASiR‐V 0%, 50%, and 100% and the Bone kernel [AR‐0, AR‐50, AR‐100; parts (a)‐(c), respectively] and ASiR‐V 0%, 50%, and 100% and the Lung kernel [AR‐0, AR‐50, AR‐100; parts (d)‐(f), respectively]. Calculations for the nonprewhitening (NPW) and the nonprewhitening with eye filter (NPWE) observers are shown. Fourier domain results are shown using both peripheral and central noise power spectra (see Figure 1b,c).

Predictions for the 120 kV protocol and low dose, but with high contrast (8 mgI/mL) and lung and bone kernels are shown in Figure 5a–f (ASiR‐V 0%, 50%, or 100% only). Again, increasing discrepancies between Fourier and spatial domain approaches can be discerned for higher iterative strength of reconstruction. The choice of a NPS based on central ROIs, which is more representative of the location of the 8 mgI/mL features, made little difference compared to using peripheral ROIs. It should be noted that determination of the central NPS is likely associated with higher uncertainty, due to the necessity of using fewer and smaller ROIs compared with the peripheral NPS (see Figure 1b,c).

**FIGURE 6:**
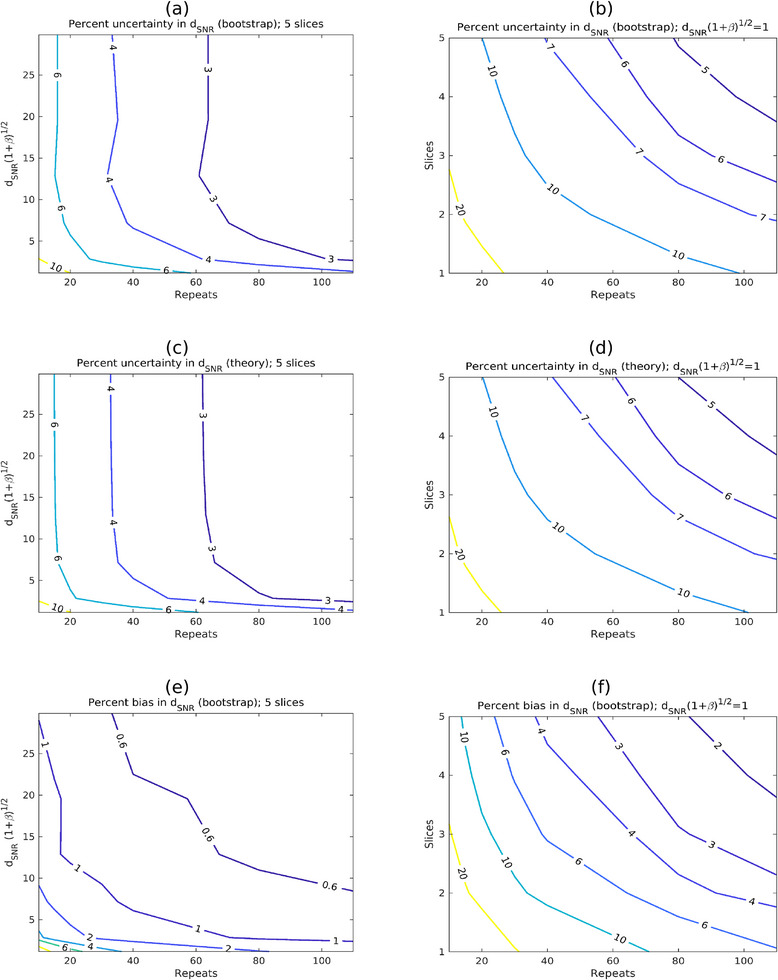
Plots in parts (a) and (b) show the percent uncertainty in detectability index (d_SNR_) derived from bootstrap resampling. Part (a) illustrates how the uncertainty varies with the magnitude of d_SNR_(1+β)^1/2^ and the number of repeat scans (with 5 slices). Part (b) illustrates how the uncertainty varies with number of slices and number of repeats, for fixed d_SNR_(1+β)^1/2 ^= 1. Parts (c) and (d) show the same quantities, derived theoretically using Equation (24). Parts (e) and (f) present equivalent plots to (a) and (b), but with bootstrap estimates of percentage bias in d_SNR_ instead of uncertainty.

Figure 6a,b and 6c,d present percent uncertainties in *d*_SNR_ estimates, calculated using bootstrap techniques and Equation (24), respectively. The data used for bootstrap sampling corresponded to CTDI_vol _= 5 mGy with 2 mgI/mL, reconstructed with ASiR‐V 0%. However, from Equation (24), the percent uncertainty is only expected to depend on the number of slices, repeats, and the detectability index itself [or on $d_{SNR}{\left(1+\beta \right)}^{1/2}$, where β is the coefficient of internal noise]. The results are consistent with this expectation. The plots are nearly identical for bootstrap and theory, confirming the validity of the derived formula. Figure 6e,f present equivalent plots to Figure 6a,b, but for the estimated bias from the bootstrap calculations. The smallest feature in the scans (1 mm) is associated with $d_{\mathrm{SNR}}{\left(1+\beta \right)}^{1/2}\approx 1$, and the largest uncertainty and bias. Even in this case, for 5 slices and 100 repeat scans, these are below 5% and 2%, respectively.

## 4 DISCUSSION

A practical methodology has been proposed for determining the detectability index of nonprewhitening observers in CT, using only the repeated imaging of a phantom, ROI placements, and simple manipulations with images. As the methodology is performed in the spatial domain, it does not rely on noise stationarity, the contrast‐independence of resolution and noise, or on symmetry of spatial resolution and noise properties in the axial plane. It also does not require explicit determination of the noise covariance matrix, which has been utilized in some spatial domain approaches.^10^ A formula has been provided for the uncertainty that can be expected on a determination of detectability index.

The results indicate that for the reconstructions where linearity and noise stationarity are expected to be most strongly maintained (i.e., ASiR‐V 0%), the typical Fourier domain calculation strategy and the spatial domain method closely correspond, regardless of whether an eye filter is applied. For strong iterative strength in reconstruction (ASiR‐V) and for DLR reconstruction (TrueFidelity), there were some discrepancies between Fourier and spatial approaches, with the Fourier‐based method estimating higher detectability to a small to moderate extent. Interestingly, recent calculations for an ideal (prewhitening) observer and iterative reconstructions (including ASiR‐V) showed the opposite trend.^13^ The implication of the current study is, however, that the advantages of strong noise suppression, whether through iterative reconstruction or DLR, might be exaggerated when adopting a Fourier domain approach to NPW model observers. A previous work, investigating a different scanner and reconstruction algorithm, found agreement between Fourier domain calculations of NPWE and human performance for filtered backprojection, but an overestimation of performance for iterative reconstruction.^11^ It is interesting to consider whether such a discrepancy would have been reduced if the NPWE observer had been calculated in the spatial domain. Further investigation with comparisons to human observers is warranted.

Our preferred interpretation of the current results is to acknowledge the usefulness of the Fourier‐domain approach, but to recognize that it should be used judicially and the interpretations of its results made with caution. This is not a new insight. It is well‐acknowledged, for example, that the detectability calculated in a Fourier‐based approach in some sense represents an average over spatial locations, rather at a specific point in an image.^7^, ^20^, ^29^, ^35^ A contribution of this article is the provision of a practical method that can be used for assessing quantitatively the applicability of a Fourier approach for a given feature, phantom, protocol, and reconstruction.

In addition to use as a tool for validation of typical Fourier‐based approaches, the proposed spatial‐domain method has the advantage of simplicity, requiring only repeat scanning of a phantom, ROI placements, and simple mathematical operations on images. It obviates the necessity of the calculation of the NPS and TTF while also avoiding the need for the noise covariance matrix to be determined. These properties lend themselves to routine implementation as part of controls in a quality assurance program. These advantages come at the price of the approach only being applicable to the calculation of detectability for features physically realizable in a phantom.

This work utilized a single cylindrical phantom with two flat contrast levels (2 and 8 mgI/mL) in a uniform background. Signal and background characteristics in a patient are more irregular and complex, as is the behavior of nonlinear reconstruction algorithms.^7^, ^15^, ^16^, ^36^, ^37^, ^38^ For example, iterative denoising using certain priors (e.g., total variation) might perform better with simple piecewise constant phantoms than in patients, while a DLR algorithm trained exclusively on patient data, might perform sub‐optimally on simple phantoms.^38^ For clinically relevant assessments, more anthropomorphic phantoms are required for realistic task‐based assessments. Fortunately, advances in 3D printing technology have made this feasible.^39^ The necessary complex lesions and backgrounds constitute a challenge for Fourier domain methods, where, for example, the 2D TTF determination typically assumes circular symmetry and the NPS is determined in a uniform background. The challenges may not be insurmountable.^36^ However, the spatial domain methodology presented here can immediately be applied to more complex scenarios, with only modifications to ROI definitions and the treatment of the background subtraction.

This work only constituted a demonstration of the proposed spatial domain approach and was limited in scope. Additional limitations were the use of only two single‐energy protocols (80 and 120 kV) and two dose levels (CTDI_vol_ = 5 and 13 mGy). Further, acquisitions were on a single scanner (GE Revolution), with the iterative reconstruction and DLR algorithms for a single vendor used (ASiR‐V and True Fidelity), and limited reconstruction kernels investigated (Standard, Lung, and Bone for for ASiR‐V and Standard for DLR). Comparisons of spatial domain and Fourier domain results for nonprewhitening observers for a wider arrange of features, scanners, protocols, and reconstruction algorithms would be of interest, as would comparisons to human observers.

## 5 CONCLUSION

A practical spatial‐domain approach is proposed for the calculation of nonprewhitening model observers in CT. The methodology relies on repeated imaging and ROI placement and is demonstrated to agree with Fourier‐domain calculations for linear‐like CT reconstruction algorithms. Moderately higher detectability was observed for the Fourier‐domain approach, for iterative reconstruction with strong iterative strength and for a deep learning based reconstruction algorithm (< 10% NRMSD for the nonprewhitening model observer). In addition to the method being a valuable tool for validation of typical Fourier‐based methodologies, it lends itself to the routine calculation of detectability for features embedded in a phantom.

## CONFLICT OF INTEREST STATEMENT

The authors have no relevant conflicts of interest to declare.

## Data Availability

The data that support the findings of this study are available from the corresponding author upon reasonable request.

## APPENDIX A ALGORITHM FOR SPATIAL DOMAIN CALCULATION OF NONPREWHITENING OBSERVERS

### A.1

Figure 1d illustrates the phantom with the features and the associated ROIs (see ROI_1_, ROI_2_, and ROI_b_). In the following description, an “ROI” should be interpreted as a mask identifying pixels of interest. An “ROI image” should be interpreted as the background‐subtracted pixel values corresponding to an ROI mask in a particular image instance. Such an ROI image may be with a feature present (i.e., ROI_2_) or absent (i.e., ROI_1_). An “ROI template” is the expected values of the ROI image with a feature present, approximated as an average over a set of ROI images. We consider an image set of *n* repeats scans with *m* usable slices per scan. Calculations involve the following steps:

1. Generate an averaged image by taking the mean over all slices and repeats;
2. Locate the centers of the features of interest in the averaged image (e.g., 1, 2, 4, 6, 8, 10 mm);
3. Create feature ROIs (i.e., ROI_2_) centered on the features, with radii corresponding to the feature radius expanded by 4 pixels;
4. Create background ROIs (i.e., ROI_b_) as annuli surrounding the features (between 8 and 12 pixels beyond the feature radii);
5. Calculate the background value, *b*, for each feature, in each slice, using the mean value in the appropriate background ROI, averaged over the *n* image instances per slice;
6. Create noise ROIs (i.e., ROI_1_), of identical size to the feature ROIs, but in the uniform surrounding material away from any feature (e.g., located at the equivalent left‐right mirror positions);
7. For each feature, in the feature present (ROI_2_) and feature absent (ROI_1_) conditions, calculate the *n* x *m* background‐subtracted ROI images, using the slice‐specific background value, b; see Equation 14;
8. Estimate the ROI template for each feature as the averaged feature‐present ROI image; see Equation 15; a single template should be calculated for each feature by averaging over slices;
9. Iterate through the *n* x *m* image instances, calculating the decision variable for each feature under the feature present and absent conditions (${L_{\mathrm{NPW}}|}_{h=2}$ and ${L_{\mathrm{NPW}}|}_{h=1}$), using the ROI templates and the ROI images; see Equation 16;
10. For each of the features, in each of the *m* slices, calculate: (i) the mean decision variable (Equation 17) and (ii) the variances for its feature present (ROI_2_) and absent (ROI_1_) cases (Equation 18);
11. Calculate the average of the decision variable (i.e., signal) and of its variances over the *m* slices;
12. Using the slice‐averaged signal and variance, calculate d_SNR_ for each feature—see Equation 20;

Several points regarding the implementation should be noted. First, all images available for a set of repeats were used to create the average template, with a slice‐specific background subtraction. This was to suppress as much noise as possible in the template. For accurate calculations, the noise in the template estimate must be low. As we have included every image in the template estimation, strictly the decision variable is nonlinear, and the noise in the template is correlated to the noise in each slice. However, for a sufficient number of slices and repeats, the consequences are negligible.

Second, ROI positioning is not critical, if the size of the ROI is large enough to comfortably encompass the feature, including not just its size at input but after the degradation due to the spatial transfer of the imaging system. However, it is advised to not make the ROIs unnecessarily large, to avoid any accumulation of numerical errors.

Third, the noise ROIs were placed at the same displacement as the feature ROI, but at the mirror position on the left side. As the phantom shape has left‐right symmetry, it was assumed that this would represent the signal‐absent condition, even when noise characteristics depart from stationarity. Therefore, in Equation 18, rather than acquiring a separate set of images with the signal absent, it was assumed that $i_{j,h=1}^{s}=i_{j^{\prime },h=2}^{s}$, where *j’* represents a pixel in the mirror location (i.e., ROI_1_).

Fourth, for the calculation of the NPWE observer, an identical process was carried out as for the NPW observer, but with images pre‐filtered with the eye‐filter (Equation 12). The whole (zero‐padded) image was Fourier transformed in MATLAB, multiplied by the filter and then inverse transformed. To reduce undesirable effects in the vicinity of air gaps, CT numbers lower than ‐100 HU were set to 0 HU prior to transforming the images. An alternative approach would have been to Fourier transform reduced cut‐out regions around the ROIs, avoiding the inclusion of air regions.

Fifth, “signals” and “variances” were averaged over slices prior to calculating d_SNR_, rather than averaging d_SNR_ for each slice. This results in a lower bias in the estimate of detectability index.

## APPENDIX B UNCERTAINTY IN DETECTABILITY INDEX

### B.1

Consider a scenario with *m* slices and *n* repeat scans per slice. The signal and noises averaged over slices can be determined as $\bar{S}=\frac{1}{m}\;\sum_{i=1}^{m}S_{i}$, $\bar{N_{1}^{2}}=\frac{1}{m}\;\sum_{i=1}^{m}N_{1,i}^{2}$, and $\bar{N_{2}^{2}}=\frac{1}{m}\;\sum_{i=1}^{m}N_{2,i}^{2}$, respectively. The decision variable SNR is,

(B1)
$$\mathrm{SNR}={\left(\frac{1}{1+\beta }\frac{{\left(\bar{S}\right)}^{2}}{\left(\frac{1}{2}\bar{N_{1}^{2}}+\frac{1}{2}\bar{N_{2}^{2}}\right)}\right)}^{1/2},$$

where, for generality, we have included the coefficient of internal noise, β. Propagation of uncertainties then provides the result,

(B2)
$$\mathrm{Var}\left[\mathrm{SNR}\right]={\mathrm{SNR}}^{2}\left(\frac{\mathrm{Var}\left[\bar{S}\right]}{{\left(\bar{S}\right)}^{2}}+\frac{\mathrm{Var}\left[\bar{N_{1}^{2}}\right]+\mathrm{Var}\left[\bar{N_{2}^{2}}\right]}{4{\left(\bar{N_{1}^{2}}+\bar{N_{2}^{2}}\right)}^{2}}\right).$$

One option for determining the variances on the left‐hand‐side of Equation (B3) is to calculate the standard errors for $\bar{S}$, $\bar{N_{1}^{2}}$ and $\bar{N_{2}^{2}}$, based on the values of S, $N_{1}^{2,}$ and $N_{2}^{2}$ in the *m* slices. A convenient alternative, however, is to insert theoretical results. Assuming independent sampling among slices and repeats,

(B3)
$$\mathrm{Var}\left[\bar{S}\right]=\frac{1}{nm}\;{\bar{N}}^{2},$$

(B4)
$$\begin{array}{ccc} \mathrm{Var}\left[\bar{N_{1}^{2}}\right] & = & \frac{2}{\left(n-1\right)m}{\left(\bar{N_{1}^{2}}\right)}^{2}\;\text{and}\;\\ & & \mathrm{Var}\left[\bar{N_{2}^{2}}\right]=\frac{2}{\left(n-1\right)m}{\left(\bar{N_{2}^{2}}\right)}^{2}. \end{array}$$

Result (B4) follows from the definitions of a signal and its variance and the central limit theorem. The results (B5) follow from a standard formula for the variance of a variance^40^ and the central limit theorem. Therefore, using Equations (B1), (B2), (B3), (B4), (B5),

(B5)
$$\begin{array}{ccc} \mathrm{Var}\left[\mathrm{SNR}\right] & = & \frac{1}{\left(1+\beta \right)}\frac{1}{nm}+\frac{1}{4}\frac{1}{\left(n-1\right)m}\\ & & \times \;{\mathrm{SNR}}^{2}\left(2\frac{{\left(\bar{N_{1}^{2}}\right)}^{2}+{\left(\bar{N_{2}^{2}}\right)}^{2}}{{\left(\bar{N_{1}^{2}}+\bar{N_{2}^{2}}\right)}^{2}}\right) \end{array}$$

In the situation that the variances in the signal present and absent conditions are similar, they can be approximated as equal and Equation (B6) simplifies to,

(B6)
$$u=\sqrt{\mathrm{Var}\left[\mathrm{SNR}\right]}=\sqrt{\frac{1}{\left(1+\beta \right)}\frac{1}{nm}+\frac{1}{4}\frac{1}{\left(n-1\right)m}{\mathrm{SNR}}^{2}}$$

This provides an estimate of the uncertainty (*u*) in the detectability index ($d_{\mathrm{SNR}}=\mathrm{SNR}$), assuming one signal ROI and one noise ROI per image, per feature.
